# Role of Sociodemographic Variables in the Association Between Physical Performance and Balance Disorders in Older Adults

**DOI:** 10.1155/jare/9826332

**Published:** 2026-08-16

**Authors:** Ximena A. Salazar-Gutarra, Steffano A. Valle-Farfán, Yumaira Chacon, Omar R. Rodriguez, José F. Parodi, Alvaro M. Ñaña-Cordova, Fernando M. Runzer-Colmenares

**Affiliations:** ^1^ Human Medicine Program, Faculty of Health Sciences, Científica del Sur University, Lima, Peru; ^2^ Abbott Healthcare Costa Rica, San José, Costa Rica; ^3^ Abbott Laboratories, Lima, Peru, abbott.com; ^4^ Center of Aging Research (CIEN), Faculty of Human Medicine, San Martín de Porres University, Lima, Peru; ^5^ CHANGE Research Working Group, Human Medicine Program, Científica del Sur University, Lima, Peru

**Keywords:** aged, aging, geriatric assessment (MeSH terms), physical fitness, postural balance

## Abstract

**Background:**

Aging is associated with decreased physical and cognitive functions, increasing the risk of balance disorders and falls in older adults. Physical performance is a key early indicator of frailty and disability, and balance disorders impair the ability to carry out daily living activities. This study aimed to determine whether sociodemographic factors modify the association between physical performance and balance disorders among older adults in Peru.

**Methods:**

A cross‐sectional analytical study was conducted using secondary data from two cohorts: the CEMENA Frailty Study (*n* = 1896) and the ANDES‐FRAIL Study (*n* = 453). After excluding participants with incomplete data, 2200 older adults aged ≥ 60 years were analyzed. Physical performance was measured using the Short Physical Performance Battery (SPPB), and balance disorders were assessed with the functional reach test (FRT). Crude and adjusted Poisson regression models stratified by sociodemographic variables were performed, adjusting for multimorbidity and functional dependence.

**Results:**

Of the participants, 52.09% had impaired physical performance, and 44.05% exhibited balance disorders. Impaired physical performance was significantly associated with balance disorders. Associations were observed among individuals aged ≥ 80 years (PR: 2.72; 95% CI: 2.23–3.32), men (PR: 2.59; 95% CI: 2.22–3.04), those with < 11 years of education (PR: 2.43; 95% CI: 2.00–2.95), and coastal residents (PR: 2.63; 95% CI: 2.32–2.99). The strongest associations were observed among adults aged ≥ 80 years and coastal residents after adjustment for multimorbidity and functional dependence.

**Conclusion:**

Physical performance impairment is strongly associated with balance disorders among older adults, and this relationship is influenced by sociodemographic factors. These findings suggest that sociodemographic characteristics should be considered when evaluating the relationship between physical performance and balance disorders in older adults.

## 1. Introduction

The World Health Organization (WHO) indicates that global life expectancy has improved to reach 80 years or more [1]. In Peru, older adults aged 60 years or older represent 12.4% of the population, exceeding 4 million individuals [2]. A study conducted among older Peruvians living in extreme poverty reported a 17.3% prevalence of disability in activities of daily living (ADLs) and highlighted major barriers to healthcare access, with more than 60% of participants not receiving preventive health services [3]. Disability and functional decline may reduce intrinsic capacity and negatively affect mobility and independence in older adults [3]. In addition, factors, such as age, education, social network, pain, and depression, have been associated with lower physical activity levels in aging populations [4].

Age‐related physiological decline contributes to multimorbidity, frailty, and deterioration of physical function [5, 6]. Frailty is characterized by reduced strength, endurance, and physiological reserve, increasing vulnerability to falls, disability, and dependency [7]. Furthermore, the coexistence of frailty, possible sarcopenia, and malnutrition has been associated with cognitive impairment and functional dependence [8]. Older adults also frequently experience balance disorders related to age‐associated motor and sensory decline, which may impair the performance of ADLs and increase fall risk [9].

Physical performance refers to the ability to perform physical activities efficiently and is considered an early indicator of frailty, disability, and sarcopenia in older adults [10, 11]. Gait and balance disorders are recognized predictors of falls [12], and previous studies have shown that lower physical activity levels and poorer physical performance are associated with increased fall frequency [13]. Likewise, balance disorders have been associated with reduced ability to perform ADLs [14]. Age‐related declines in gait speed, lower limb muscle strength, proprioception, vestibular function, and reaction time may simultaneously impair postural control and physical performance [14–16].

Although previous studies have evaluated physical performance and falls separately, evidence regarding how sociodemographic characteristics influence the association between physical performance and balance disorders in Peruvian older adults remains limited. This paper seeks to identify whether the relationship between physical performance and balance disorders is different based on sociodemographic factors in older people living in coastal and high‐Andean areas of Peru. Previous studies derived from the CEMENA Frailty study and the ANDES‐FRAIL study have evaluated frailty, mobility, and functional outcomes in Peruvian older adults [17, 18].

## 2. Methods

### 2.1. Study Design

The study followed an observational, retrospective, analytical, and cross‐sectional design using secondary data from the CEMENA Frailty Study [17] and the ANDES‐FRAIL Study [18]. Both cohorts applied standardized geriatric assessments and comparable operational definitions for physical performance, balance disorders, and covariates, which allowed a secondary analysis using harmonized variables. However, potential methodological heterogeneity between cohorts cannot be completely excluded. To explore the consistency of findings, stratified and sensitivity analyses according to cohort‐related variables were performed. The manuscript was prepared following STROBE recommendations. The completed STROBE checklist is available as Supporting File S1.

### 2.2. Study Population

The study population was made up of participants from the CEMENA Frailty Study and ANDES‐FRAIL Study. The CEMENA Frailty Study was done from 2010 to 2016 at CEMENA in Callao, Peru. It was a sample of 1,896 participants aged 60 years and above who were recruited to determine the determinants of frailty. Outpatients were included, but patients who were in hospital, home care, HIV/AIDS, severe functional disability, or severe dementia or who refused consent were excluded.

The ANDES‐FRAIL Study was conducted from 2013 to 2020 and included 453 older adults aged 60 years and older from 12 rural and urban communities located in the Peruvian Andes at elevations above 2500 m above sea level. People with severe dementia or disability were excluded. Both studies employed nonprobabilistic sampling.

**FIGURE 1:**
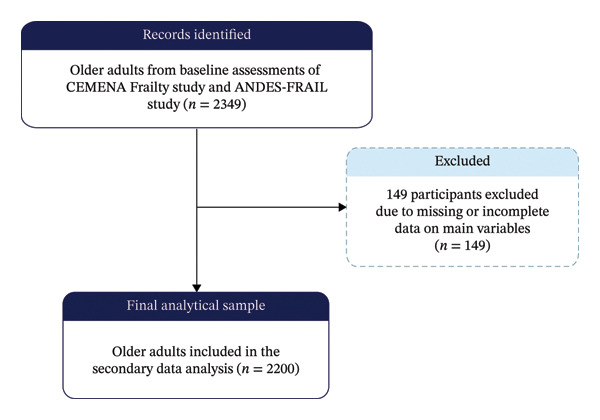
Flow diagram for population selection in this study.

At first, the data of 2349 participants were used. We dropped data from 149 participants because they had insufficient sociodemographic data (age: *n* = 2, sex: *n* = 3, education level: *n* = 94, living alone: *n* = 6) or missing covariates (physical performance: *n* = 1, balance disorders: *n* = 43). The final sample comprised 2200 participants (Figure 1).

### 2.3. Study Variables

The primary variables were physical performance and balance disorders, assessed using the short physical performance battery (SPPB) and the functional reach test (FRT), respectively.

The SPPB is a widely used and validated instrument for assessing physical performance in older adults [19]. It has three components which include standing balance, gait speed, and the ability to sit and lie down from a chair, scored on a 12‐point scale (0 = *severe limitation*; 12 = *minimal limitation*) [20].

The FRT, a widely used screening tool for assessing balance impairment in older adults, was employed to evaluate balance disorders in this study [21]. A cutoff point of < 20.32 cm was used to define impaired balance capacity [22]. Given the heterogeneity of FRT thresholds reported in the literature, the cutoff selected for this study was based on previous analyses conducted in the ANDES‐FRAIL cohort, which included Peruvian older adults with demographic and clinical characteristics similar to those of the present study population [22]. The use of this locally derived threshold enhances the clinical relevance of the assessment and facilitates comparability with previous studies conducted in Peruvian older adults. Participants stood upright with feet shoulder‐width apart and were instructed to reach forward maximally without stepping. Reach distance was measured in centimeters using the dominant arm.

Sociodemographic variables included sex, age, educational level (≤ 11 years; > 11 years), living alone (yes or no), and region (coast and highlands).

Covariates included comorbidities (hypertension, diabetes, lumbar osteoarthritis, obesity, and chronic obstructive pulmonary disease). The presence of two or more conditions was termed as multimorbidity; having one or none was termed as no multimorbidity [23]. Comorbidities were obtained from medical records and/or self‐report during structured geriatric interviews. The Barthel Index was used to determine the level of dependence on ADLs and a score of 95 or lower [24].

### 2.4. Statistical Analysis

Descriptive statistics were reported as frequencies and percentages. Bivariate analysis was performed using the chi‐square test. Two Poisson regression models were constructed: a crude model and an adjusted model. The adjusted model included multimorbidity and functional dependence and was stratified according to sociodemographic variables (age, sex, living arrangement, region, and educational level). Prevalence ratios (PRs) and 95% confidence intervals (95% CIs) were estimated. This approach was selected because it provides direct estimates of PRs for binary outcomes and is recommended for cross‐sectional studies, particularly when outcome prevalence is not low. To explore potential heterogeneity related to the origin of the participants, stratified analyses were performed comparing the Andean and coastal cohorts. Similar associations were observed across strata, suggesting that cohort‐specific characteristics did not materially affect the study findings. Model assumptions were evaluated by assessing the mean–variance relationship and verifying log‐linearity for the dependent variable. Multicollinearity was assessed using the variance inflation factor (VIF), considering values < 5 acceptable. To evaluate the robustness of the findings and address potential heterogeneity between cohorts, sensitivity analyses were performed through stratified analyses according to region and cohort‐related population characteristics. However, formal multilevel analyses or fixed‐effects models by cohort were not performed, which should be considered when interpreting the results. All analyses were conducted using Stata Version 18.0. To account for potential heterogeneity between cohorts, sensitivity analyses were performed by fitting multivariable Poisson regression models including cohort (ANDES‐FRAIL and CEMENA) as a fixed effect. These analyses were conducted to evaluate whether between‐cohort differences influenced the association between physical performance and balance disorders. The robustness of the findings was assessed by comparing PRs and pseudo *R* ^2^ values across model specifications.

### 2.5. Ethical Norms

The present study is a secondary analysis of two publicly available and fully anonymized databases (ANDES‐FRAIL and CEMENA). Both original cohort studies were approved by the Ethics Committee of the Peruvian Naval Medical Center. The ANDES‐FRAIL Study was approved under Memorandum No. 49 (April 21, 2009), and the CEMENA Study was approved under Memorandum No. 54 (May 30, 2011). Written informed consent was obtained from all participants in the original studies. Because the databases are coded, anonymized, and publicly available, no additional informed consent was required for the present secondary analysis.

## 3. Results

Table 1 summarizes the characteristics of the study population. Among the 2,200 older adults included, 44.05% (*n* = 969) presented balance disorders according to the FRT, while 52.09% (*n* = 1146) exhibited impaired physical performance based on the SPPB. Most participants were aged 60–79 years (60.77%), male (54.64%), had ≥ 11 years of education (55.45%), and resided in the coastal region (79.55%). Additionally, 37.18% of participants presented multimorbidity and 66.23% showed functional dependence in ADLs (Table 1).

**TABLE 1:** Descriptive analysis of sociodemographic variables and covariates. (*n* = 2200).

Variables	*n*	%
*Age*		
60–79 years	1337	60.77
≥ 80 years	863	39.23

*Sex*		
Female	998	45.36
Male	1202	54.64

*Educational level*		
< 11 years	980	44.55
≥ 11 years	1220	55.45

*Living alone*		
No	1871	85.05
Yes	329	14.95

*Region*		
Highland	450	20.45
Coast	1750	79.55

*Comorbidities*		
0‐1	1382	62.82
≥ 2	818	37.18

*Functional dependence (Barthel)*		
Normal	743	33.77
Functional dependence	1457	66.23

*Physical performance (SPPB)*		
Normal	1054	47.91
Altered	1146	52.09

*Balance disorder (functional reach test)*		
Normal	1231	55.95
Balance disorder	969	44.05

Abbreviation: SPPB, short physical performance battery.

Table 2 presents the bivariate association between sociodemographic variables, impaired physical performance, and balance disorders. Impaired physical performance was significantly associated with balance disorders across all sociodemographic strata (*p* < 0.001). The highest proportion of balance disorders among participants with impaired physical performance was observed in adults aged ≥ 80 years (78.26%), individuals with < 11 years of education (81.82%), and residents from highland regions (82.10%). Similar proportions were observed between women (74.73%) and men (72.35%), as well as between participants living alone (72.17%) and those not living alone (73.65%) (Table 2).

**TABLE 2:** Bivariate analysis of sociodemographic variables, physical performance, and balance disorders (*n* = 2200).

Variables	Functional reach test normal *n* (%)	Functional reach test altered *n* (%)	*p* value^∗^
60–79 years			
Normal SPPB	508 (66.93)	172 (29.76)	< 0.001
Altered SPPB	251 (33.07)	406 (70.24)	
≥ 80 years			
Normal SPPB	289 (61.23)	85 (21.74)	< 0.001
Altered SPPB	183 (38.77)	306 (78.26)	
Female			
Normal SPPB	338 (62.71)	116 (25.27)	< 0.001
Altered SPPB	201 (37.29)	343 (74.73)	
Male			
Normal SPPB	459 (66.33)	141 (27.65)	< 0.001
Altered SPPB	233 (33.67)	369 (72.35)	
Educational level (< 11 years)			
Normal SPPB	272 (57.38)	92 (18.18)	< 0.001
Altered SPPB	202 (42.62)	414 (81.82)	
Educational level (≥ 11 years)			
Normal SPPB	525 (69.35)	165 (35.64)	< 0.001
Altered SPPB	232 (30.65)	298 (64.36)	
Living alone			
Normal SPPB	128 (59.81)	32 (27.83)	< 0.001
Altered SPPB	86 (40.19)	83 (72.17)	
Does not live alone			
Normal SPPB	669 (65.78)	225 (26.35)	< 0.001
Altered SPPB	348 (34.22)	629 (73.65)	
Living in the highlands			
Normal SPPB	103 (46.61)	41 (17.90)	< 0.001
Altered SPPB	118 (53.39)	188 (82.10)	
Living on the coast			
Normal SPPB	694 (68.71)	216 (29.19)	< 0.001
Altered SPPB	316 (31.29)	524 (70.81)	

Abbreviation: SPPB, short physical performance battery.

^∗^ *p* values calculated using the test of *χ* ^2^.

Table 3 shows the association between covariates and the main study variables. Functional dependence was significantly associated with both balance disorders and impaired physical performance (*p* = 0.013 and *p* = 0.001, respectively). In contrast, multimorbidity was associated with balance disorders (*p* = 0.002) but not with impaired physical performance (*p* = 0.110) (Table 3).

**TABLE 3:** Bivariate analysis of covariates with physical performance and balance disorders (*n* = 2200).

Variables	Functional reach test normal	Functional reach test altered	*p* value^∗^	Normal SPPB	Altered SPPB	*p* value^∗^
Functional dependence			0.013			0.001
No	443 (35.99)	300 (30.96)		394 (37.38)	349 (30.45)	
Yes	788 (64.01)	669 (69.04)		660 (62.62)	797 (69.55)	
Comorbidities			0.002			0.110
0‐1	738 (59.95)	644 (66.46)		644 (61.10)	738 (64.40)	
≥ 2	493 (40.05)	325 (33.54)		410 (38.90)	408 (35.60)	

Abbreviation: SPPB, short physical performance battery.

^∗^ *p* values calculated using the test of *χ* ^2^.

Table 4 presents the crude and adjusted Poisson regression models stratified by sociodemographic variables. After adjustment for multimorbidity and functional dependence, impaired physical performance remained consistently associated with balance disorders across all strata. The strongest associations were observed among participants aged ≥ 80 years (aPR: 2.72; 95% CI: 2.23–3.32), men (aPR: 2.59; 95% CI: 2.22–3.04), individuals with < 11 years of education (aPR: 2.43; 95% CI: 2.00–2.95), and coastal residents (aPR: 2.63; 95% CI: 2.32–2.99) (Table 4).

**TABLE 4:** Crude and adjusted Poisson regression analysis by strata of sociodemographic variables to determine the association between physical performance and balance disorders (*n* = 2200).

Variables	Crude model prevalence ratio (95% confidence interval)	Adjusted model^∗^ prevalence ratio (95% confidence interval)
60–79 years		
Normal SPPB	Reference	Reference
Altered SPPB	2.44 (2.12–2.82)	2.40 (2.08–2.77)
≥ 80 years		
Normal SPPB	Reference	Reference
Altered SPPB	2.75 (2.26–3.36)	2.72 (2.23–3.32)
Female		
Normal SPPB	Reference	Reference
Altered SPPB	2.47 (2.08–2.92)	2.46 (2.08–2.92)
Male		
Normal SPPB	Reference	Reference
Altered SPPB	2.61 (2.23–3.05)	2.59 (2.22–3.04)
Educational level (< 11 years)		
Normal SPPB	Reference	Reference
Altered SPPB	2.66 (2.21–3.20)	2.43 (2.00–2.95)
Educational level (≥ 11 years)		
Normal SPPB	Reference	Reference
Altered SPPB	2.35 (2.02–2.74)	2.31 (1.98–2.69)
Living alone		
Normal SPPB	Reference	Reference
Altered SPPB	2.46 (1.74–3.47)	2.45 (1.73–3.46)
Does not live alone		
Normal SPPB	Reference	Reference
Altered SPPB	2.56 (2.26–2.89)	2.53 (2.24–2.86)
Living in the highlands		
Normal SPPB	Reference	Reference
Altered SPPB	2.16 (1.64–2.84)	1.83 (1.34–2.49)
Living on the coast		
Normal SPPB	Reference	Reference
Altered SPPB	2.63 (2.31–2.99)	2.63 (2.32–2.99)

Abbreviation: SPPB, short physical performance battery.

^∗^Model adjusted for comorbidities and functional dependence variables.

Additionally, formal interaction analyses were conducted to evaluate whether the association between impaired physical performance and balance disorders differed according to sociodemographic characteristics. Significant interaction effects were observed for age (*p* = 0.032), sex (*p* = 0.034), educational level (*p* = 0.001), living arrangement (*p* = 0.048), and region (*p* = 0.002) suggesting that the magnitude of the association between impaired physical performance and balance disorders varied across these population subgroups (Table 5).

**TABLE 5:** Interaction analysis between impaired physical performance and sociodemographic variables in relation to balance disorders among older adults (*n* = 2200).

Interaction term	PR	95% CI	*p* value
SPPB × age	1.13	1.01–1.27	0.032
SPPB × sex	1.15	1.04–1.17	0.034
SPPB × educational level	1.19	1.08–1.26	0.001
SPPB × living alone	1.21	1.14–1.25	0.048
SPPB × region	1.24	1.15–1.29	0.002

*Note:* Models adjusted for multimorbidity and functional dependence.

Abbreviations: PR, prevalence ratio; SPPB, short physical performance battery.

As part of the sensitivity analyses, multivariable Poisson regression models accounting for cohort effects were fitted (Table 6). The association between altered physical performance and balance disorders remained consistent across all sociodemographic strata. Older adults with altered SPPB scores had a higher prevalence of balance disorders regardless of age group, sex, educational level, living arrangement, or geographic region. The magnitude of the associations was similar to those observed in the primary analyses, suggesting that the inclusion of cohort effects did not substantially modify the study findings and supporting the robustness of the results. Furthermore, only minor differences were observed in the pseudo *R* ^2^ values across models (Table 4: 0.234; Table 5: 0.203; and Table 6: 0.256), supporting the robustness of the findings and suggesting that between‐cohort differences had minimal influence on the observed associations.

**TABLE 6:** Sensitivity analysis using multivariable Poisson regression including cohort fixed effects to evaluate the association between physical performance and balance disorders among older adults from combined Peruvian cohorts (*n* = 2200).

Variables	Adjusted model^∗^ prevalence ratio (95% confidence interval)
60–79 years	
Normal SPPB	Reference
Altered SPPB	2.33 (2.09–2.45)
≥ 80 years	
Normal SPPB	Reference
Altered SPPB	2.32 (2.12–3.01)
Female	
Normal SPPB	Reference
Altered SPPB	2.27 (2.03–2.77)
Male	
Normal SPPB	Reference
Altered SPPB	2.11 (1.92–2.66)
Educational level (< 11 years)	
Normal SPPB	Reference
Altered SPPB	2.43 (2.01–2.91)
Educational level (≥ 11 years)	
Normal SPPB	Reference
Altered SPPB	2.37 (1.91–2.65)
Living alone	
Normal SPPB	Reference
Altered SPPB	2.45 (1.71–3.46)
Does not live alone	
Normal SPPB	Reference
Altered SPPB	2.13 (2.19–2.77)
Living in the highlands	
Normal SPPB	Reference
Altered SPPB	1.82 (1.34–2.47)
Living on the coast	
Normal SPPB	Reference
Altered SPPB	2.22 (2.02–2.61)

Abbreviation: SPPB, short physical performance battery.

^∗^Model adjusted for comorbidities and functional dependence variables.

## 4. Discussion

This study evaluated the association between impaired physical performance and balance disorders according to sociodemographic characteristics among older adults living in coastal and highland regions of Peru. Our findings showed that impaired physical performance was consistently associated with balance disorders after adjustment for multimorbidity and functional dependence across all evaluated strata, including age, sex, educational level, living arrangement, and region. Interaction analyses suggest that the association between physical performance and balance disorders may vary according to sociodemographic and clinical characteristics. Therefore, FRT results should not be interpreted in isolation, but rather in conjunction with individual patient characteristics to improve risk stratification and guide personalized preventive interventions.

Older adults aged ≥ 80 years showed the strongest association between impaired physical performance and balance disorders. These findings are consistent with a Colombian study reporting higher frailty risk and reduced muscle strength among adults older than 75 years [25]. Similarly, previous evidence has shown that better physical performance acts as a protective factor against falls, particularly in advanced age groups [26]. Age‐related declines in muscle strength, proprioception, gait stability, and neurological function could be associated with deterioration in both balance and physical performance. Additionally, multimorbidity and functional dependence may coexist with greater mobility limitations in the oldest‐old population.

Regarding sex differences, impaired physical performance was associated with balance disorders in both men and women; however, slightly higher estimates were observed among men after adjustment for multimorbidity and functional dependence. Previous studies have reported sex‐related differences in physical performance and fall risk among older adults [27, 28]. However, the present study was not designed to evaluate the mechanisms underlying these differences, and these findings should be interpreted cautiously.

Lower educational attainment was also associated with a higher prevalence of balance disorders among participants with impaired physical performance. Similar findings have been reported in Indonesian older adults, where lower education, aging, memory impairment, and arthritis were associated with increased fall risk [29]. Educational level may reflect broader social and health‐related conditions associated with aging [30, 31]. However, evidence regarding the relationship between educational level and falls remains inconsistent, as some systematic reviews have found limited or no direct association [32].

In relation to living arrangement, impaired physical performance was associated with balance disorders regardless of whether participants lived alone or accompanied, although slightly higher estimates were observed among those living alone. Previous studies have reported associations between living arrangement and physical performance among older adults [33, 34]. However, the variable “living alone” does not necessarily reflect social isolation, loneliness, or social support, and therefore, its interpretation should be made cautiously.

Regional differences were also identified. Although impaired physical performance was associated with balance disorders in both highland and coastal populations, the strongest association was observed among coastal residents. Previous studies conducted in Peruvian high‐Andean populations have reported differences in physical performance according to geographic setting [35, 36]. However, the present study was not designed to determine the factors underlying these regional differences.

The findings of this study highlight the importance of evaluating physical performance and balance disorders simultaneously in older adults, particularly among vulnerable sociodemographic groups. Balance disorders are multifactorial conditions influenced by musculoskeletal, sensory, and functional factors, and previous studies have highlighted the relevance of multidimensional geriatric assessment in aging populations [37, 38].

The present study combined data from two Peruvian cohorts that employed comparable eligibility criteria and standardized assessment procedures. Although differences in recruitment settings and population characteristics may have introduced some heterogeneity, sensitivity analyses incorporating cohort effects yielded estimates similar to those observed in the primary analyses. These findings suggest that between‐cohort differences had minimal impact on the association between physical performance and balance disorders.

This study has several limitations. First, its cross‐sectional design precludes causal inference between impaired physical performance and balance disorders. Second, both cohorts used nonprobabilistic sampling methods, which may limit external validity and the representativeness of the findings. Third, although multimorbidity was considered in the adjusted analyses, individual chronic conditions were not evaluated separately.

Additionally, several potentially relevant variables, including socioeconomic status, physical activity, cognitive status, nutritional status, medication use, family support, environmental barriers, and healthcare access patterns, were unavailable in the databases. Consequently, residual confounding cannot be ruled out despite adjustment for available covariates. Missing data and participant exclusions may also have introduced selection bias if the probability of missingness differed according to participant characteristics or outcomes. Although sensitivity analyses demonstrated stable estimates across different model specifications, the potential influence of unmeasured factors cannot be completely excluded and should be considered when interpreting the findings.

Furthermore, potential heterogeneity between cohorts may have influenced the results. Standardized geriatric assessments and harmonized variables were used but the differences in recruitment settings, geographic regions, and healthcare catchment areas may have introduced variability between cohorts. However, sensitivity analyses incorporating cohort effects yielded estimates similar to those observed in the primary analyses, suggesting that between‐cohort differences had minimal impact on the observed associations.

Finally, the FRT and the balance component of the SPPB assess related aspects of postural control, creating a degree of conceptual overlap that may have strengthened the observed associations. Therefore, part of the relationship identified may reflect convergent measurement of similar functional domains rather than entirely independent constructs.

Despite these limitations, the study also has important strengths. It included a large sample of older adults from both coastal and highland regions of Peru and used validated instruments to assess physical performance and balance disorders. In addition, the analyses were adjusted for relevant geriatric and clinical variables, allowing a broader evaluation of the observed associations across different sociodemographic groups [39, 40].

## 5. Conclusion

Our findings show that impaired physical performance is consistently associated with balance disorders among Peruvian older adults across multiple sociodemographic groups. In particular, stronger associations were observed among adults aged ≥ 80 years, men, individuals with lower educational attainment, and coastal residents. Future longitudinal studies are needed to clarify causal pathways and evaluate whether interventions targeting physical performance may contribute to improving balance outcomes and reducing fall risk in older populations.

## Author Contributions

Conceptualization: Ximena A. Salazar‐Gutarra, Steffano A. Valle‐Farfán, Yumaira Chacon, Omar R. Rodriguez, Fernando M. Runzer‐Colmenares, José F. Parodi, and Alvaro M. Ñaña‐Cordova.

Data curation: Ximena A. Salazar‐Gutarra, Steffano A. Valle‐Farfán, Yumaira Chacon, Omar R. Rodriguez, Fernando M. Runzer‐Colmenares, José F. Parodi, and Alvaro M. Ñaña‐Cordova.

Methodology: Fernando M. Runzer‐Colmenares, José F. Parodi, and Alvaro M. Ñaña‐Cordova.

Investigation: Ximena A. Salazar‐Gutarra, Steffano A. Valle‐Farfán, Yumaira Chacon, and Omar R. Rodriguez.

Supervision: Fernando M. Runzer‐Colmenares and José F. Parodi.

Writing–original draft: Ximena A. Salazar‐Gutarra, Steffano A. Valle‐Farfán, Yumaira Chacon, and Omar R. Rodriguez.

Writing–review and editing: Fernando M. Runzer‐Colmenares, José F. Parodi, and Alvaro M. Ñaña‐Cordova.

## Funding

No funding received for this manuscript.

## Conflicts of Interest

The authors declare no conflicts of interest.

## Supporting Information

Additional supporting information can be found online in the Supporting Information section.

## Supporting information


**Supporting Information** Supporting File S1. STROBE checklist for cross‐sectional studies describing the reporting items addressed in this manuscript.

## Data Availability

The data that support the findings of this study are openly available in OSFHOME at https://osf.io/rsc7q/ and in Figshare at https://doi.org/10.6084/m9.figshare.13059011.v2.
